# Environmental DNA-Based Ecological Risk Assessment of PAHs in Aged Petroleum-Contaminated Soils

**DOI:** 10.3390/toxics13050357

**Published:** 2025-04-29

**Authors:** Jinrong Huang, Chang Zhou, Fanyong Song, Tianyuan Li, Jianing Wang, Xiaowen Fu

**Affiliations:** Shandong Provincial Key Laboratory of Applied Microbiology, Ecology Institute, Qilu University of Technology (Shandong Academy of Sciences), Jinan 250200, China; 10431221175@stu.qlu.edu.cn (J.H.); 13053399704@163.com (C.Z.); songfy@qlu.edu.cn (F.S.); 707027@qlu.edu.cn (T.L.); wangjn@sdas.org (J.W.)

**Keywords:** petroleum contamination, polycyclic aromatic hydrocarbons, toxic equivalency, species sensitivity distribution curve

## Abstract

(1) Background: Polycyclic aromatic hydrocarbons (PAHs) are important components of petroleum and pose a serious threat to the soil environment of oil production well sites. Therefore, scientific risk thresholds and ecological risk assessment methods must be established for PAHs in petroleum-contaminated soils. (2) Methods: In this study, based on the environmental DNA (eDNA) method, the soil bacterial community was considered as a receptor to assess the ecological risks of PAH contamination in aged petroleum-polluted soils. A combination of the risk quotient and the equivalent toxicity factor was used to assess the ecological risk of PAHs. (3) Results: A dose–response curve was plotted to determine the 50% effective concentration (EC_50_) of the total equivalent toxicity for 16 PAHs (∑TEQ_BaP_) in petroleum-contaminated soils. Following the plot of the species sensitivity distribution (SSD) curve, the hazardous concentration for protecting 95% species values (HC_5_) of petroleum hydrocarbons (TPHs), electrical conductivity (EC), and total equivalent toxicity of PAHs were calculated to be 892.1 μs·cm^−1^, 149.9 mg·kg^−1^, and 0.2601 mg·kg^−1^, respectively. The regression models of the distribution factor (DF) and aging factor (AF) were defined as DF = −1.132 SOM + 0.033PAHs + 9.968 and AF = 242.518 SOM + 1256.029 lgpH + 0.024 EC − 1415.447. Following calibrations of the DF and AF, the value of HC_5_ was determined as 0.1956 mg·kg^−1^, which could be considered the risk threshold of the total toxicity of PAHs. The calibrated toxicity data distribution was consistent with that of the normal cumulative probability distribution model. The results showed that 50% of the aged petroleum-contaminated soils showed high-risk levels of bacterial communities exposed to PAHs. (4) Conclusions: This study provides a reference for deriving the ecological risk threshold of soil pollutants and explores alternative methods for the ecological risk assessment of PAHs at specific sites.

## 1. Introduction

China is the second-largest consumer of petroleum globally and presents abundant resources and a high production of domestic petroleum [1,2]. Petroleum-contaminated soil is formed over time, resulting in a notable decrease in soil nutrient availability, microbial activity, and other indicators, thereby affecting the material circulation of the soil ecosystem. Polycyclic aromatic hydrocarbons (PAHs) represent one of the main components of petroleum and are key pollutants that exhibit carcinogenicity, teratogenicity, and mutagenicity. The United States Environmental Protection Agency (EPA) has listed 16 types of PAHs as priority pollutants for control, which are the types of PAHs we commonly refer to [3,4]. Wang et al. [5] used absorption–distribution–metabolism–excretion–toxicity (ADMET) to calculate and predict the physicochemical properties and toxicity of 19 PAHs released from petroleum, but this method is highly dependent on the species toxicity database. Ghobakhloo et al. [6] conducted a health risk assessment of PAHs from oil spills based on Monte Carlo simulation and the United States Environmental Protection Agency (USEPA) model. This method is more convenient than traditional methods, but there are still uncertainties in the results. As the current ecological risk assessment system for PAHs is not sufficiently standardized, there is an urgent need to develop risk assessment methods for polycyclic aromatic hydrocarbons, especially in soil media [7]. Due to the complexity of the soil environmental system and complex environmental behaviors of PAHs, such as adsorption, migration, and transformation, establishing a standardized ecological risk assessment method for PAHs in oil-contaminated soils remains challenging [8,9].

The species sensitivity distribution (SSD) method constructs cumulative distribution curves by mathematically fitting toxicity data for different species of a pollutant to determine a safe concentration in the ecosystem, expressed as HC_5_, which is the lowest concentration at which 5% of species are adversely affected or 95% of species are protected [10,11,12]. This method is based on the assumption that the species are randomly selected and can represent the community structure of a given ecosystem [13]. Currently, the SSD method is widely used for ecological risk assessments because of its direct principles, simple calculations, and ease of application. For instance, Deng et al. [14] used the SSD approach to estimate the ecological safety standard levels of BaP under different land uses, providing a basis for the lack of ecological risk assessments in soil. Kwak et al. [15] used the SSD to derive the harmful concentration of phenol, a common petroleum contaminant in soil. The results can be used for the establishment of phenol standards or strategies for protecting the terrestrial environment. However, the SSD method is highly dependent on the quality and quantity of data, and differences in experimental conditions may lead to variations in the sensitivity of the receptor species [16]. Moreover, SSD is typically based on toxicity data for single pollutants and does not completely account for the complexity of ecosystems, where interactions among multiple pollutants in real-world environments can increase the uncertainty of the SSD results [17]. Bioinformatic methods, which are rapidly developing, have been integrated to address the limitations of traditional SSD techniques, thus leading to the proposal of the environmental DNA (eDNA) SSD method. eDNA technology identifies the presence and abundance of target species by detecting DNA in environmental samples (such as water, soil, and air), thereby assessing biodiversity and species distribution [18,19]. Raiz et al. [20] were the first to combine the eDNA monitoring of environmental biodiversity with SSD models and evaluate the toxic effects of pollutants on different species, thereby providing a novel approach for ecological risk assessment based on eDNA. Huang et al. [21] combined environmental DNA with SSD to comprehensively evaluate the composite contaminated soil with aniline, petroleum hydrocarbons, etc. as environmental stress factors, and derived the ecological risk values of each environmental factor. Above all, the introduction of eDNA technology, with its high detection rate for low-abundance species, has enhanced the sensitivity and accuracy of community analysis, playing a crucial role in the derivation of pollutant thresholds and advancing the field of ecological risk assessment.

Contributing to the diversity of PAHs in soil and the unclear mechanisms of their interactions, the toxicity equivalence factor of Benzo[a]pyrene (BaP) [22], which is defined as dimension 1 due to its high toxicity, wide presence, deeply studied toxicity mechanism, and significant position, has been used to convert various PAHs into equivalent toxicities (TEQ), with toxicity thresholds expressed in terms of the toxicity equivalents [23]. In this study, considering the influence of differences in the physicochemical properties and microbial community compositions of contaminated sites, the sensitivity of test species to the pollutants varied; thus, the toxicity values from the existing toxicity databases were not suitable for studying the toxicity of PAHs in soils experiencing long-term petroleum contamination. Therefore, for the first time, dose–response curves, constructed based on the changes in microbial abundance with pollutant concentrations to determine the EC_50_ and SSD curves, were used to calculate the HC_5_. Additionally, as the soil is subjected to dual stresses from salinity-alkalinity and PAH contamination, soil aging and adsorption–desorption effects significantly influence the migration and transformation of PAHs in the soil. Thus, the data were calibrated using aging factors (AFs) calculated by the ratio of TEQ to the original TEQ under different aging times and distribution factors (DFs), which was calculated based on the phase equilibrium, and the risk threshold of PAHs was calculated by combining the eDNA and SSD methods. The risk quotient (RQ) method combined with the relative toxicity coefficients was used to calculate the RQ values and evaluate the risk levels of contaminated soils. The results presented herein provide a reference for determining the ecological risk threshold of soil pollutants and generating alternative methods for the ecological risk assessment of PAHs at specific sites.

## 2. Materials and Methods

### 2.1. Soil Samples

**Figure 1:**
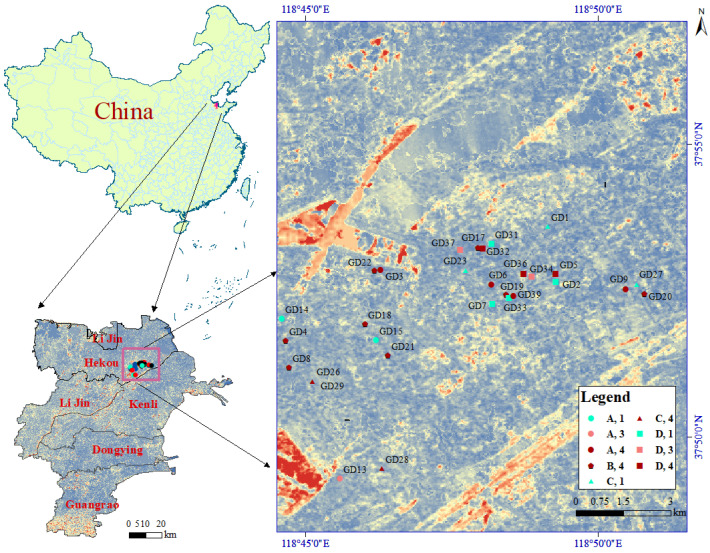
Maps of the sampling points of aged petroleum-contaminated soils.

This study focused on aged petroleum-contaminated soils from abandoned soils in the Shengli Oilfield, which is situated in a geographically distinctive area within the Yellow River Delta region, Dongying, Shandong Province, China. This area has been subjected to long-term petroleum contamination, and the soil is characterized by saline-alkali properties, leading to a higher probability of soil pollution risks. A total of 32 soil samples that were 20 cm below the surface soil with varying degrees of aged petroleum-contamination were collected from the study site by the quarter method based on the previous investigation of the aging time and the latitude and longitude coordinates of the oil well. Each sample was divided into two portions: one was sealed in a polytetrafluoroethylene (PTFE) bag and stored at 4 °C for the analysis of the soil physicochemical properties, whereas the other was preserved in sterile centrifuge tubes and stored at −80 °C for 16S amplicon sequencing, three repetitions for each part. In order to judge the preliminary investigations of the soil surrounding the oil wells, the concentrations of 16 PAHs, electric conductivity, pH, and total petroleum hydrocarbons (TPHs) were determined. The sampling points are shown in the ArcGIS satellite map in Figure 1.

### 2.2. Quantitative Analysis of PAHs and TPHs

The PAH analysis was conducted according to the method described by Antoaneta [24]. Soil samples were pretreated and analyzed for 16 priority-controlled PAHs using gas chromatography-mass spectrometry (GC-MS) (GCMS-TQ8050 NX, SHIMADZU, Kyoto, Japan). PAHs were identified based on the retention time and mass spectrometry, and quantification was performed using the internal standard method to determine the concentrations of PAHs. Qualitative analysis was performed using the retention time and mass spectrometry, whereas quantitative analysis was conducted using the internal standard method [25].

Petroleum hydrocarbons (C10–C40) in the soil were extracted using the Soxhlet extraction method [26]. Following the standard method for the determination of petroleum hydrocarbons (C10–C40) in soil (HJ 1021-2019) [27], GC (GC 6890N, Agilent, Santa Clara, CA, USA) was used for the quantitative analysis and detection of petroleum hydrocarbons in the soil.

### 2.3. Soil Microbial Community Analysis

Soil DNA was extracted using a soil DNA extraction kit and direct amplification kit (Tsingke Biotechnology, Beijing, China). DNA was lysed from the soil using a lysis buffer and the extraction process was conducted by Shanghai Personal Biotechnology Co. Ltd., Qingdao Branch (Qingdao, China). Following total DNA extraction, DNA was quantified using 1% agarose gel electrophoresis. The DNA samples that passed quality control were stored at −20 °C for further use. A 20 μL PCR reaction system was prepared, consisting of 2 μL of 1 × T3 Mix buffer, 0.8 μL each of forward and reverse primers (5 μmol/L), 0.4 μL of r Taq polymerase, and 10 ng of DNA template. The reaction volume was adjusted to 20 μL using ultrapure sterile water. After PCR amplification, the products were stored at −4 °C. The V3–V4 region of the 16S rRNA gene was amplified using the primers 338F (5′-ACTCCTACGGGAGGCAGCA-3′) and 806R (5′-GGACTACHVGGGTWTCTAAT-3′). The amplification process was conducted by Shanghai Personal Biotechnology Co., Ltd. (Shanghai, China). The amplified products were subjected to gel electrophoresis and the target bands were excised for library construction. The DNA fragments of the bacterial and fungal communities were sequenced using the Illumina platform in the paired-end mode. The paired-end reads were processed through assembly, filtering, and noise reduction to obtain high-quality sequences. The DADA2 method was used for primer removal, quality filtering, denoising, assembly, and chimera removal, resulting in unique sequences referred to as amplicon sequence variants (ASVs). Taxonomic annotation of the representative sequences for each ASV was performed using the classify-sklearn algorithm in QIIME2, ultimately yielding species information corresponding to each sequence.

### 2.4. Data Calibration

The total equivalent toxic concentrations of the 16 PAHs (TEQ_BaP_) [28] were calibrated based on their distribution in the soil and aging process. PAHs exhibit complex and frequent migration and transformation pathways in the soil, and their distribution plays a dominant role. Therefore, the Karickhoff empirical formula [29,30,31,32,33] was used in this study to calculate the distribution coefficient, and then the concentration of ΣTEQ_BaP_ in the soil was recalculated to complete the calibration [34]. The distribution factor (DF) is the ratio of the calibrated concentration value to the original concentration data. A regression model was built according to the environmental factors of each soil. In addition, because the collected soil was a long-term oil-aged soil, the effect of aging on the behavior of PAHs in the soil environment could not be ignored. Within the aging experiment, the experimental group that did not undergo substrate addition was designated as the ‘blank control’. The experimental group that underwent the addition of 10 mg·kg^−1^ of 16 PAHs was used in accordance with the stipulated requirements of the International Organization for Standardization (ISO 22030-2005) [35] as well as under the simulated outdoor conditions. AF is the ratio of the equivalent toxicity concentration at different aging periods to the original data. The calculation formula for the distribution effect and specific operation of the aging experiment design are detailed in the Supplementary Materials.

### 2.5. Data Analysis and Statistics

#### 2.5.1. Screening of Sensitive and Tolerant Species

Sensitive and tolerant species are defined as those whose relative abundance significantly decreases or increases, respectively, with changes in the soil pollutant concentrations. Based on the relationship between the species relative abundance and pollutant concentration, univariate linear regression was performed using R language. Significance at *p* < 0.1 was used as the criterion for screening sensitive or tolerant species. Specifically, when the regression coefficient (R) was >0, the species was considered tolerant, whereas when R < 0, the species was classified as sensitive.

#### 2.5.2. Construction of SSD Curves and Calculation of Ecological Risk Thresholds

The eDNA-SSD method is based on changes in the relative abundance of biological species to construct SSD curves, ultimately calculating the HC_5_ for different pollutants. First, the dose–response curves for each species under different pollutant concentrations were fitted using GraphPad Prism 9 software, and the EC_50_ was calculated. The formula used for this calculation is as follows [21]:

(1) $$y=\frac{y_{0}}{1+e^{\left(b\left(x-m\right)\right)}}$$

where:

y: Relative index (%);

x: Logarithmic concentration of total equivalent toxicity, mg·kg^−1^;

y_0_ and b: Fitting parameters;

m: Natural logarithm of ECx.

The relative index (%) represents the ratio of the response of the test indicators at other concentrations to that of the CK, where the CK test indicators are set at 100%. In this study, x was set as 50.

The toxicity data of the species were sorted in ascending order and imported into the data fitting of the SSD curve. The model corresponding to the minimum root mean square error (RMSE) value among the fitted values was considered the optimal model. When constructing an SSD curve, the commonly used fitting functions include normal and logistics. The parameter equations for each model are as follows:

Normal model [36]:

(2) $$f\left(x\right)=\frac{1}{x\sigma \sqrt{2\pi }}e^{-\frac{{\left(x-\mu \right)}^{2}}{{2\sigma }^{2}}}$$

where:

μ: Average level of sensitivity to pollutants of all species involved in the analysis;

σ: Standard deviation;

x: Concentration of pollutants.

Logistics model [37,38]:

(3) $$f\left(x\right)=\frac{e^{-\left(x-\alpha \right)/s}}{s{\left(1+e^{-\left(x-\alpha \right)/s}\right)}^{2}}$$

where:

*x*: Susceptibility of a species to contaminants or environmental stressors while representing EC_50_ in this research;

*α*: Median of species sensitivity;

s: Degree of dispersion of logistic distribution.

Finally, the ecological risk threshold (HC_5_) for 95% of the protected species was calculated based on the SSD curves of the set. The parametric equation is as follows:

(4) HCq=b1q1k−11c

where HC_(q)_ represents the pollutant content causing ecological risks to q% of the species, measured in mg·kg^−1^ (HC_5_ was adopted in this paper). Based on this formula, the corresponding ecological risk thresholds for different pollutants can be calculated.

#### 2.5.3. Risk Level Evaluation

The toxicity of different PAH monomers varies. Evaluating the overall toxicity risk of PAHs is not a simple addition of the mass fractions of PAH monomers. To quantify the comprehensive toxicity risk level, this study adopted toxic equivalency factors (TEFs) for the calculation [39].

To evaluate the ecological risks of PAHs in petroleum-contaminated soil collected from the oil production plant, this study adopted the risk entropy value method proposed by Kalf et al. [40] combined with the relative toxicity coefficient and used the negligible concentrations (NCs) and maximum permissible concentrations (MPCs) to calculate the RQ values of PAHs. The formulae are as follows:

RQ_NCs_ = c_i_/C_Qv(NCs)_ (5)

RQ_MPCs_ = c_i_/C_Qv(MPCs)_ (6)

RQ∑16_PAHs (MPCs)_ = ∑(i = 1)^16 RQ_MPCs_ (7)

where:

RQ_NCs_: The minimum risk entropy value of target substance i;

RQ_MPCs_: The maximum risk entropy value of target substance i;

C_i_: The mass fraction of target substance i in soil, mg·kg^−1^;

C_QV(NCs)_: The minimum risk standard value, mg·kg^−1^;

C_QV(MPCs)_: The maximum risk standard value, mg·kg^−1^.

The classification criteria for the ecological risk levels of the PAHs are listed in Table 1.

#### 2.5.4. Biological Data Processing and Plotting

Data were processed using Origin 2024. A *p* ≤ 0.05 was considered statistically significant. The α-diversity of the soil microbial community was analyzed using the Shannon and ACE indices to evaluate the complexity of species diversity under different remediation treatments. These indices were calculated using QIIME software (version 1.7.0) and visualized using R software (version 2.15.3). To assess the differences in species complexity among the different remediation measures, beta diversity was calculated using QIIME software (version 1.9.1) and analyzed. The DF and AF were determined by comparing the ratio of raw data to toxicity data before and after the distribution/aging effects. Data processing and multiple regression analyses were conducted using SPSS software (version 26). Dose–response and SSD curves were fitted using GraphPad Prism 9.5. Based on the fitting results, appropriate models were selected to calculate the ecological risk thresholds.

## 3. Results and Discussion

### 3.1. Analysis of Soil Physical and Chemical Properties and Biological Distribution

#### 3.1.1. Analysis of Soil Physical and Chemical Properties

The physicochemical properties of the collected soil samples are summarized in Table 2. The results indicated that all soil samples were weakly alkaline to alkaline saline-alkali soils, with pH values ranging from 7.9 to 8.9. This is consistent with the findings of previous studies [41] on petroleum-contaminated soils in the Shengli Oilfield. The organic matter content ranged from 3.685% to 11.735%, which is consistent with the results reported by Liu et al. [42]. The TPH content ranged from 0 to 588.1 mg·kg^−1^, which is significantly lower than the concentration range of 500–15,000 mg·kg^−1^ reported in previous studies [43]. This discrepancy could be attributed to the fact that earlier studies focused on soils from active oil wells, where fresh petroleum mixtures resulted in higher TPH concentrations. Conversely, the soils collected in this study underwent aging for 10–40 years, during which the petroleum components were significantly reduced due to volatilization, natural degradation, photodegradation, and microbial activity. All soil samples exhibited varying degrees of PAH contamination, with the ΣPAH concentrations ranging from 0.6825 to 134.95 mg·kg^−1^. These PAH concentrations were low compared with the concentration range of 2.5–120 mg·kg^−1^ reported by Liu et al. [44] for contaminated soils in oil-well areas. This phenomenon can be explained by the aging process of petroleum, which leads to the adsorption, migration, and transformation of PAHs in soil, resulting in a reduction in their extractable amounts. The toxic equivalent concentration of ∑TEQ_BaP_ ranged from 0.00275 to 1.11279 mg·kg^−1^, with 25% of the samples exceeding the standard limits referring to the Maliszewska–Kordybach classification standard [45]. Under the dual stress of salinity-alkalinity and PAHs, the soil texture became compacted and presented larger particle sizes, indicating a complex organic–inorganic composite pollution scenario.

#### 3.1.2. Analysis of Relative Abundance and Biodiversity

**Figure 2:**
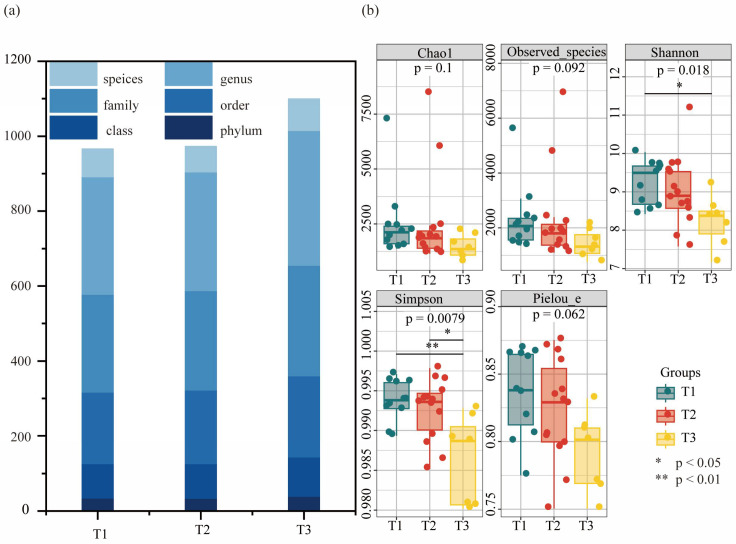
Number of taxa and diversity analysis. (**a**) Differences in the number of classification units at different toxicity concentrations. (**b**) Analysis of α-diversity.

The number of taxa in the petroleum-contaminated soil with different aging times under the seven classification levels is shown in Figure 2a. Groups T1, T2, and T3 showed the taxonomic levels of species in oil-impregnated soils with aging times of 0–20, 20–30, and 30–50 years, respectively. No significant difference was observed in the number of biological species in the soils aged 0 to 30 years, although the number of species in soils aged 30 years and above increased significantly. This was due to the toxic effect of petroleum components on microorganisms in the soil during the early stages of aging (within 10 years) and the associated negative impact on the biological community. When petroleum is exposed to the external environment for a long time (10–30 years), the gum in petroleum becomes closely bound to the soil. Consequently, the amount of oxygen in the soil cannot be maintained, and increased microbial respiration activities lead to a decrease in the biological classification level. After 30 years of soil aging, the biological community in the soil had adapted to the oil-polluted environment and some dominant species had multiplied. Coupled with the adsorption and degradation of petroleum components, the soil exhibited breathing space, and more microorganisms participated in the polluted soil, resulting in an increase in the number of biological classification levels.

The results of the α-diversity analysis, as shown in Figure 2b, indicate that changes in the total toxicity of PAHs significantly affected the α-diversity of the soil microbial communities. As the equivalent toxicity increases, the richness, diversity, and evenness of soil microorganisms decrease. The Kruskal–Wallis (K–W) test results revealed significant differences in the diversity indices (Shannon and Simpson) (*p* < 0.05), with the species diversity significantly decreasing as the TEQ_BaP_ concentration increased. High PAH toxicity exerts a pronounced disturbance on the soil microbial communities, demonstrating toxic effects on microorganisms and accelerating the decline of species.

**Figure 3:**
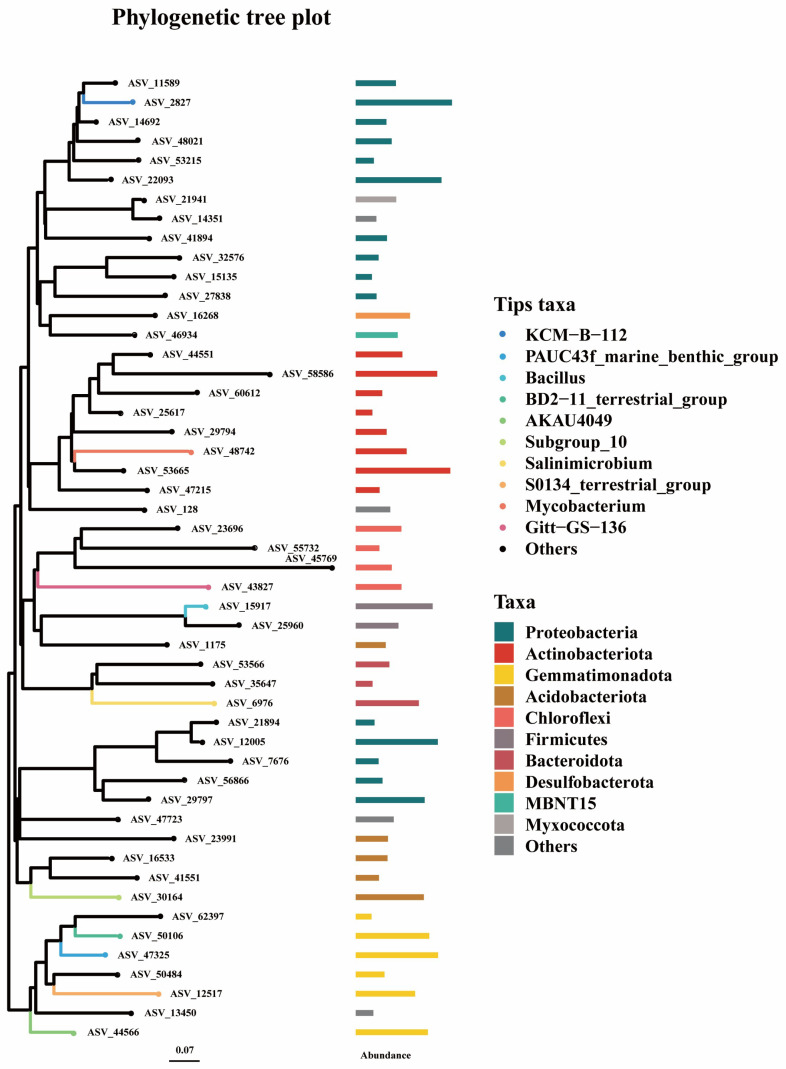
Composition and distribution of soil biological communities.

The taxonomic composition analysis of the collected soil samples showed that the dominant biological groups with high relative abundance affected by the degree of soil aging at the gate level were Proteobacteria, Actinobacteriota, Gemmatimonadota, Acidobacteriota, and Chloroflexi (Figure 3). The dominant species with high relative abundance at the genus level were *KCM-B-112*, *PAUC43f_marine_benthic_group*, *Bacillus*, and *BD2-11 terrestrial*. The degree of soil fertilization did not affect the overall composition of the soil microbial community, although it affected the distribution of microorganisms. Jiang et al. [46] found that changing the aging time of petroleum had a significant perturbing effect on the soil microbial community. The specific changes vary according to the different physical and chemical properties of the soil, especially salinity and hydrocarbon content. Yerulker et al. [47] found that as the aging process progressed, physical and chemical characteristics of the soil were altered, giving rise to variations in the community structure and abundance of microorganisms. The results of this study demonstrate that there are significant differences in the abundance of microbial communities. The underlying cause may be attributed to the prolonged aging time of the collected soil, which had an average age of up to 20 years, coupled with the predominance of primary oil-tolerant strains among the existing microorganisms. This resulted in minimal variations in the composition of the microbial communities. It has been demonstrated that changes in soil properties have a significant impact on the growth ability of microorganisms. These changes can be attributed to the altered environmental conditions, which in turn result in considerable differences in community abundance.

### 3.2. Identification of Key Stress Factors and Screening of Sensitive/Tolerant Species

#### 3.2.1. Identification of Key Stress Factors

**Figure 4:**
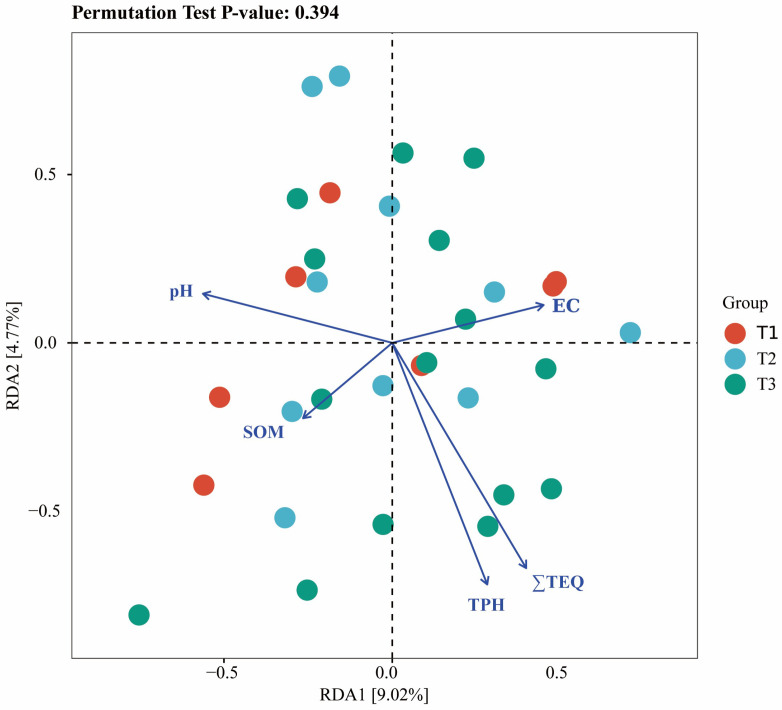
Redundancy analysis of microorganisms with respect to the environmental factors.

The results indicated that the key stress factors that significantly affected the soil biological communities were ∑TEQ_(BaP)_, TPH, and EC. Redundancy analysis (RDA) was performed using the relative abundance of soil species and the listed stress factors, as shown in Figure 4. The RDA results revealed that the total explanatory power of environmental factors for the distribution differences in the soil microbial communities was 13.79%, which effectively explained the variation in microbial distribution. Specifically, RDA1 and RDA2 accounted for 4.77% and 9.02% of the variance, respectively. The variation in soil biological communities was most significantly influenced by ∑TEQ_(BaP)_, whereas the distribution of biological communities was affected to a certain degree by TPH, pH, and EC.

**Figure 5:**
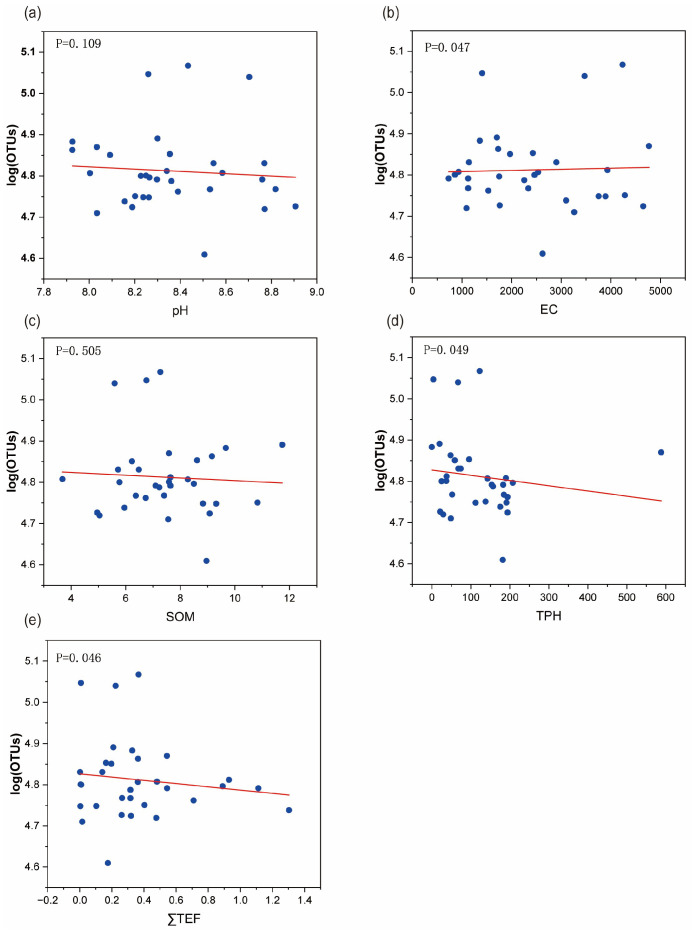
Linear regression of OTUs and environmental factors. (**a**–**e**) Monadic linear regression between the number of OTUs and pH, EC, SOM, TPH and ∑TEQ_BaP_, respectively. P denotes the significance level of the univariate regression equation between the environmental factors and the number of OTUs.

Univariate linear regression analysis was conducted using SPSS software to examine the relationship between the number of operational taxonomic units (OTUs) and the five environmental factors, as illustrated in Figure 5. The results demonstrated that TEQ, TPH, and EC significantly affected the distribution and abundance of the soil biological communities (*p* < 0.05). As the concentrations of these environmental factors increased, the number of OTUs decreased, indicating an inhibitory effect on the growth of soil microorganisms and suggesting toxic effects on the microbial community. Therefore, these factors were identified as the key environmental stressors in the soil samples collected from oil production sites. The parameter data for each environmental factor are presented in the Supplementary Materials.

The primary environmental stress factors identified in this study area, namely ∑TEQ_BaP_, TPH, and EC, were found to exert a substantial inhibitory effect on the growth and reproduction of microorganisms in the soil. This, in turn, led to a reduction in their functional activities, thereby impeding the process of microbial degradation of PAHs. This process led to a further exacerbation of the degree of pollution by PAHs in the aged petroleum soil. Research has demonstrated that shifts in biological communities have a detrimental effect on soil nutrient cycling, organic matter decomposition, and plant health, resulting in ecological imbalance [48,49]. The identification of key environmental stress factors provides a more comprehensive set of monitoring parameter references for the formulation of soil remediation strategies in the subsequent stage.

#### 3.2.2. Identification of Sensitive and Tolerant Species

In this study, a univariate linear regression analysis was conducted based on changes in the relative abundance of soil microbial species to identify tolerant and sensitive species. For ∑TEQ_BaP_, one tolerant species, *Nitrospira* sp., and four sensitive species, *Desulfuromonas_Desulfuromonas* sp., *Bacterium_YC-ZSS-LKJ56*, *Halomonas xianhensis*, and *Rhodovibrio* sp., were identified. For the total petroleum hydrocarbons (TPHs), seven tolerant species were identified: *Rhodovulum*_sp., *Bacterium_YC-LK-LKJ13*, *Bacillus_hwajinpoensis*, *Halomonas_ventosae*, *Bacterium_YC-ZSS-LKJ56*, *Halomonas_xianhensis*, and *Candidatus_Tectomicrobia*. For EC, three tolerant species, *Rhodovulum* sp., *Thalassobacillus_Thalassobacillus* sp., and *Candidatus_Tectomicrobia*, and three sensitive species, *Palleronia* sp., *Actinophytocola* sp., and *Pontibacter populi*, were identified. The results are summarized in Table 3. The microbial community distribution revealed changes in the microbial community structure in the study area and reflected the pollution characteristics of the local soil. The introduction of eDNA provides a method for selecting receptors for the toxicity testing of environmental stress factors, enhancing the environmental specificity and improving accuracy.

### 3.3. Calculation of Ecological Risk Thresholds

#### 3.3.1. Ecological Risk Thresholds of Key Stress Factors

**Figure 6:**
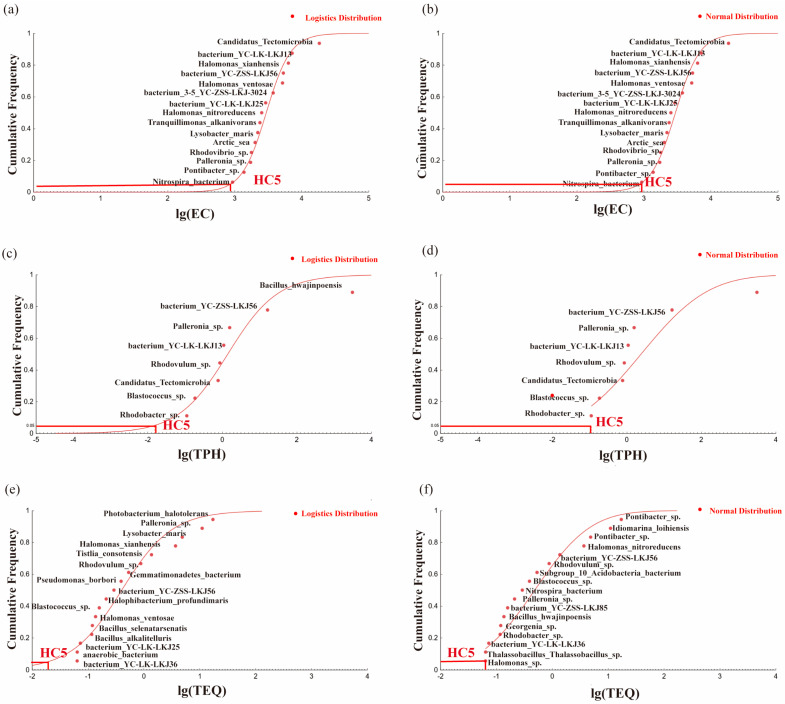
(**a**–**f**) The SSD curves fitted by different stress factors under different models.

A logistic model was used to fit the dose–response curves for the microbial community endpoints in different soils, and the EC_50_ values were calculated. The EC_50_ values for each species were sorted in ascending order, and the SSD curves were plotted based on different cumulative distribution models. The SSD curves for each model are shown in Figure 6. The RMSE was used to evaluate the goodness-of-fit of each model, with lower RMSE values indicating a better fit. The results (Table 4) demonstrate that the logistic model provided a better fit for the environmental receptors of ΣTEQ, TPH, and EC and generated smoother SSD curves and more realistic data. The calculated ecological risk thresholds for ΣTEQ, TPH, and EC were 0.0223 mg·kg^−1^, 150.0 mg·kg^−1^, and 892.072 µs·cm^−1^, respectively. The study revealed that the normal distribution model was more consistent with the collected biological data when the eDNA-SSD method was used to plot the SSD curves. The ecological risk thresholds calculated in this study were significantly lower than the current soil pollution risk control standards specified in the “Soil Environmental Quality—Risk Control Standard for Soil Contamination of Development Land” (GB36600-2018) [50]. For Class I land, the control values for BaP and TPH were 0.55 mg·kg^−1^ and 186 mg·kg^−1^, respectively, whereas for Class II land, the control values were 1.5 mg·kg^−1^ and 4600 mg·kg^−1^, respectively. The lower risk thresholds for ΣTEQ_BaP_ and TPH may be attributed to the fact that the current risk control standards are based on human health risks, whereas this study focused on the ecological risk thresholds for soil organisms [51]. Additionally, the long-term dual stress of PAH contamination and soil salinization in oilfield soils resulted in a fragile ecosystem, leading to lower ecological risk thresholds. Therefore, the current soil control standards are not suitable for ecological risk assessment in the unique habitats of oilfield soils. Furthermore, the toxicity endpoints selected in this study were species showing sensitivity to each environmental factor, and they exhibited strong responses to pollutant changes, resulting in lower calculated HC_5_ values. Particularly, because of the complex structure and active migration and transformation behavior of PAHs in soil, their ecological risk thresholds were calculated by considering multiple factors in the soil environment. Further calibration of the toxicity data is required to obtain risk thresholds specific to the target site [52].

#### 3.3.2. Data Modification

Given that PAHs in soil are significantly influenced by distribution and aging effects, the equivalent toxicity of PAHs was calibrated using DFs and AFs.

After modification, the EC_50_ was recalculated using the calibrated logarithmic total equivalent toxicity data combined with changes in the relative abundance of biological communities. The EC_50_ values before and after calibration are listed in Table 5. The results indicate that the EC_50_ increased by 2.17 to 7.10 times after accounting for the distribution effects. Furthermore, the relationship between the DF and soil properties was analyzed and established (Table 6).

The data before and after the calibration for the distribution effects and DFs were calculated. A multiple linear regression model was developed to predict the DF based on the environmental factors, as shown in Table 6. The regression equation revealed that the DF was significantly correlated with the SOM and PAH contents, exhibiting a negative correlation with the SOM and a positive correlation with the PAH content. The combined explanatory power of the SOM and PAH contents was 43.8%.

**Figure 7:**
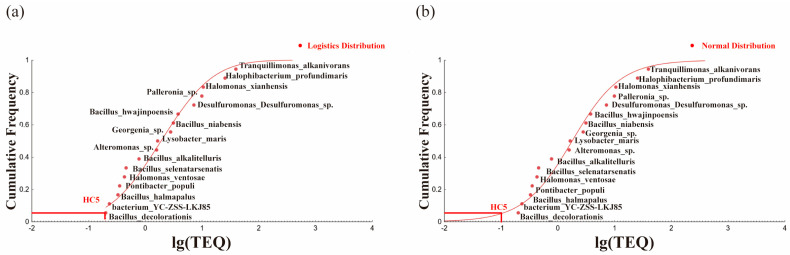
SSD curves of HC_5_ under different models after calibrating the allocation effect: (**a**) logistic distribution model; (**b**) normal distribution model.

After plotting the SSD curves, the calibrated cumulative probability distribution was observed to fit the normal model better, as illustrated in Figure 7. HC_5_ was adjusted from 0.0223 mg·kg^−1^ to 0.2005 mg·kg^−1^, demonstrating that the distribution effects significantly influenced the ecological risk threshold of the total equivalent toxicity of PAHs in soil. This calibration highlights the importance of considering the distribution and aging effects in ecological risk assessments because the species-specific responses to the total equivalent toxicity data were significantly affected by these factors.

A quantitative analysis of the contents of 16 PAHs in soil at different aging times was conducted to explore the relationship between the aging factor AF and soil environmental factors, and a regression model was established, as shown in Table 7. The PAHs’ aging factor AF was positively correlated with pH, EC, and SOM, and the three donors provided an explanation rate of 99.5%. Therefore, the degree of aging was significantly affected by the soil properties, so predictive models of AF and related soil properties were studied to correct the toxicity data that were fed back.

According to the aging factor prediction model obtained and the physicochemical properties of each sample (Table 8), the equivalent toxicity was recalculated, and the dose–effect relationship was established to obtain the EC_50_ value. The EC_50_ results before and after aging are shown in Table 7. Under the influence of aging behavior, the total equivalent toxicity EC_50_ of PAHs was increased by 3.07209~14.1436 times.

The findings indicate that the risk threshold of polycyclic aromatic hydrocarbons is considerably influenced by aging effects and distribution. Firstly, the aging process PAHs in the soil will gradually combine with the soil organic matter, thereby reducing its bioavailability and mobility [53]. Secondly, the distribution behavior of PAHs is influenced by multiple factors, including the soil type, organic matter content, pH value, and temperature, which directly affect the exposure routes and bioavailability of PAHs, and further influence their ecological risks [54]. In particular, environments such as oilfield soil present more complex risk assessments for PAHs.

#### 3.3.3. Determination of the Ecological Risk Threshold

**Figure 8:**
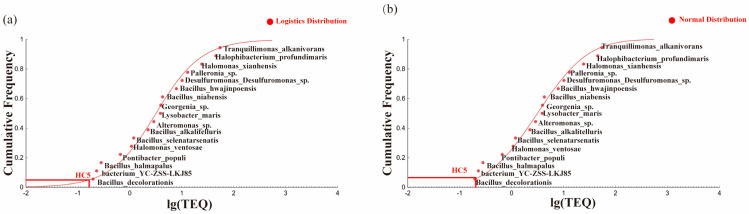
(**a**,**b**) Comparison of results of different models of the SSD curve.

Considering the influence of the distribution and aging effects on the toxicity data, the dose–response curves were refitted and the EC_50_ was recalculated. The SSD curves were plotted using different cumulative probability distribution models. The performance of the two fitting models is shown in Figure 8. The RMSE values for the normal and logistic distribution models were 0.0386 and 0.0416, respectively, indicating that the normal distribution model was more suitable for deriving the PAH thresholds in the oilfield soils. Furthermore, the value of *p* > 0.05 indicated that the fitting performance of the model was acceptable, suggesting that the model adequately described the SSD. Using the SSD curve fitted by the normal distribution model, the calculated HC_5_ was 0.1956 mg·kg^−1^, whereas the HC_5_ calculated from the uncalibrated toxicity data was 0.0223 mg·kg^−1^. The RMSE decreased, which indicated that the modified curve demonstrated a better fitting performance. The current regulatory standard for PAHs in China is 0.1 mg·kg^−1^ [50], and the value derived in this study was significantly higher than this standard. This discrepancy arose because this study converted the composite PAHs in the soil into ∑TEQ_BaP_ concentrations, which allowed for a more comprehensive consideration of the effects than generalized PAHs. Competitive inhibition and other interactions between pollutants may also occur. Moreover, the long-term exposure of oilfield soils to petroleum contamination has elevated the background levels of PAHs, leading to a higher total PAH toxicity in the soil. Furthermore, the microbial communities in these soils have developed a tolerance to PAHs due to prolonged exposure, resulting in dense microbial populations and higher ecological risk thresholds. Li et al. [55] attempted to establish soil guideline levels for several polycyclic aromatic hydrocarbons (PAHs) at typical petrochemical contaminated sites using the UK Contaminated Land Exposure Assessment Model (CLEA model) of 0.225 mg·kg^−1^ for BaP, which was similar to the values in this paper.

In summary, the introduction of eDNA for screening sensitive and tolerant species and the construction of ecological risk thresholds based on changes in microbial community abundance not only simplified the toxicity screening process, but also provided a novel approach for selecting toxicity receptors for pollutants [56]. Additionally, eDNA enabled the specific calculation of risk thresholds for the target contaminated sites, thereby facilitating ecological risk assessment processes [57]. This approach avoided the drawbacks of applying uniform regulatory standards, which could have led to a “one-size-fits-all” approach, and mitigated the risk of overprotection in certain scenarios. The methodology proposed in this study offers a new framework for deriving ecological risk thresholds for PAHs in oilfield soils and provides valuable guidance for the establishment of future regulatory thresholds.

This study characterized the distribution of microbial communities in aged petroleum-contaminated soils containing PAHs and calculated the equivalent toxicity threshold of total PAHs using the eDNA-based approach. Compared with the control values calculated for individual PAHs, this method comprehensively considered the environmental factors, biological communities, and interactions among multiple pollutants, making the derived threshold more specific and reflective of the contamination characteristics of the target assessment area. The screening of sensitive and tolerant species provided a directional basis for selecting test species and addressing the limitations of poor accuracy in the SSD curves caused by insufficient or highly variable toxicity data in toxicological databases. Moreover, it addressed the challenges of excessive data volume and overly complex threshold derivation processes. Furthermore, this study investigated the effects of aging and partitioning on the occurrence of PAHs in soils, thus providing a scientific basis for subsequent research on the pollutant thresholds in soil environments.

### 3.4. Soil Risk Assessment

The RQ method combined with BaP-equivalent toxicity was employed to assess the contamination risk of PAHs in 32 collected soil samples. In this study, the C_QV(NCs)_ value was set at 0.0028 mg·kg^−1^, whereas the C_QV(MPCs)_ value was determined using the calculated ecological risk threshold of 0.1956 mg·kg^−1^. The contamination status of each soil sample is detailed in Table 9, and the risk levels in the collected soil are shown in Figure 1. In addition, the pollution index method (PI) [58], ground accumulation index method (Igeo) [59,60], and Nemerow integrated pollution index (NIPI) [61] were added to assess the risk of the collected soil and perform comparisons with the results of this study. The results of different risk assessment methods are shown in Table 8. Details on the evaluation methods are included in the Supplementary Materials.

Among the soil samples collected from the environment surrounding the Shengli Oilfield, the ΣTEF values ranged from 0.0028 mg·kg^−1^ to 1.3034 mg·kg^−1^, with an average value of 0.3611 mg·kg^−1^. The risk assessment identified 10 low-risk sites, 3 moderate-risk sites, and 16 high-risk sites. Overall, PAH contamination in the soils surrounding the oilfield was severe, with high-risk sites accounting for 50% of the total. This highlights the urgent need for subsequent remediation decisions and the continuous monitoring of soil indicators to restore the ecological health of the oilfield soils. These results underscore the importance of addressing PAH contamination in oilfield environments, particularly given the significant proportion of high-risk sites, which require targeted remediation strategies and ongoing environmental monitoring to mitigate the long-term ecological impacts. This study revealed that partially aged soils that have undergone more than 30 years of natural aging still exhibit high-risk contamination levels. Therefore, the contamination status of these sites must be reconfirmed, and soil remediation plans developed. Human intervention measures should be implemented to remediate high-risk contaminated soils. The high risk associated with highly aged and contaminated soil may be related to the difficulty in dissolving high-ring polycyclic aromatic hydrocarbons and their low vapor pressure, which makes them recalcitrant to self-degradation activities in the soil medium and prevents their infiltration into groundwater or deep soil with wet deposition. Therefore, high-ring polycyclic aromatic hydrocarbons accumulate in the soil over a long aging period. Moreover, some polycyclic aromatic hydrocarbons derived from atmospheric deposition continue to accumulate and become enriched, resulting in a high proportion of high molecular weight polycyclic aromatic hydrocarbons (HMW-PAHs) in contaminated soil with a long aging period and high ecological risk.

The evaluation results based on the single-factor index method indicated that among the sampled soils, 26 were classified as heavily polluted, and 2 each were classified as moderately, lightly, and slightly polluted. The statistical results of the Igeo method revealed that 5 samples were classified as strongly polluted, 13, as moderately to strongly polluted, 6 as moderately polluted, and 8 as lightly to moderately polluted. The Nemerow index method classified all collected aged petroleum-contaminated soils as heavily polluted in terms of the comprehensive risk level. Although the single-factor index method presents direct calculations, it does not comprehensively account for the interactions among pollutants in the environment, leading to a 28.125% discrepancy compared with the RQ evaluation results. The Igeo method considers the influence of the soil background values and thus effectively reflects the degree of accumulation of PAHs compared with the natural background levels. Therefore, it can reveal the accumulation status of PAHs in the soil. However, the background values are subjective and overly reliant on empirical constants (K), resulting in a discrepancy of approximately 40% compared with the RQ evaluation method. Conversely, the Nemerow index method determined the comprehensive risk level of the sampled soils through a composite index and indicated that all soils were heavily polluted, thus indicating the need for remediation measures.

Comparative analyses revealed that the RQ method provided a more scientific evaluation of multiple PAHs in petroleum-contaminated soils. This method considered the interactions and uncertainties among various pollutants and offered an objective reflection of the comprehensive ecological risk in the target area. Additionally, the classification of risk levels was constrained by two conditions, thus preventing overprotection or insufficient protection scenarios.

## 4. Conclusions

This study combined environmental DNA with the relative abundance of the microbial communities’ response to changes in TEQ concentration, thereby constructing a dose–response curve and deriving the values of EC_50_ for each species. Then, the ecological risk threshold of ∑TEQ_BaP_ in the oil-aged soil surrounding the oil production well site was derived by employing the SSD method, in accordance with the toxicity data. The normal model exhibited a higher degree of congruence with the contaminated site of the aged oil well sample collected in this experiment. The initial threshold was thus determined to be 0.0223 mg·kg^−1^. In a subsequent stage of the process, the toxicity data were corrected for the effects of aging and distribution. This was followed by the determination of the HC_5_, which was calibrated to be 0.1956 mg·kg^−1^, which was a 8.8 times difference from the raw threshold. This indicates that the aging behavior and distribution effect of petroleum-contaminated soil have a significant impact on the fate of PAHs in such soil, with the consequence that the derivation of the PAH risk threshold is seriously affected.

The calculation of soil TEQs comprehensively accounts for the frequent and complex activities of different types of polycyclic aromatic hydrocarbons in soil, fully takes into account the interactions between different PAHs, highly represents the overall toxicity of polycyclic aromatic hydrocarbons, a typical pollutant, and facilitates the monitoring and management of polycyclic aromatic hydrocarbons at actual sites. In this study, the response of the relative abundance of microbial communities to the toxicity concentration of contaminants was used innovatively for the calculation of the threshold value. On the one hand, the integration of the eDNA method has led to an enhancement in the comprehensive understanding of the structural alterations of the biological community within the soil. Moreover, it has facilitated a more intuitive observation of the disturbance to the biological community caused by sudden changes in the physical and chemical properties. Conversely, an approach that integrates SSD with eDNA circumvents the suboptimal accuracy and repeatability that can result from inadequate data, or the imprecise threshold derivation that can ensue from shifts in experimental conditions attributable to excessive reliance on toxic data. Furthermore, the identification of the key stresses of the oil well site can be assisted. The site’s pollution situation should be monitored from multiple perspectives, while the ecological balance and health of the soil environment must be maintained. The soil biodiversity must be safeguarded, the bioavailability of the soil must be sustained, and the green and sustainable development of the soil must be promoted. Finally, the identification of the sensitive and tolerant species of the specific site provides a method for the selection of biological receptors, thereby rendering the receptors more representative of the site characteristics. The thresholds under scrutiny were determined through a scientific process and have been found to be persuasive by a preponderance of evidence. This study set out and analyzed the process of determining the threshold of PAHs at a contaminated site where aged petroleum has been deposited. The study provides a theoretical basis for the formulation of the PAH threshold.

At a later stage, on-site verification of the risk threshold can be advanced to verify the credibility and accuracy of the calculated risk threshold as well as its practical application range. In addition, the experiment volume of the full process monitoring of eDNA is huge, and the costs are difficult to control. It is expected that a rapid on-site detection technique of eDNA will be developed to achieve the rapid detection of biological data. Finally, this study considered only a limited number of 16 common PAH parent substances. However, polycyclic aromatic hydrocarbon derivatives are also widespread in reagent-contaminated sites. Therefore, the toxicity of PAH parent compounds and substituent derivatives should be further investigated to provide a more comprehensive assessment of the distribution and risk control of PAHs in soil.

## Figures and Tables

**Table 1:** Criteria for classifying the ecological risk levels of PAHs.

Risk Levels	∑16PAHs	
	RQ∑16PAHs_(NCs)_	RQ∑16PAHs_(MPCs)_
1	≥1, <800	>0, <1
2	≥800	>0, <1
3	<800	≥1
4	≥800	≥1

**Table 2:** Physical and chemical properties of the soil environment surrounding the oil production sites of the Shengli Oilfield.

Sample	pH	EC µs/cm	SOM %	TPH (mg/g)	∑PAHs (mg·kg^−1^)	∑TEQ_BaP_ (mg·kg^−1^)
GD1	8.51	2625.53	8.96	182.15	2.34	0.07
GD2	8.03	3261.60	7.56	48.75	16.24	0.01
GD3	8.34	3932.00	7.64	37.75	8.72	0.37
GD4	8.19	4653.40	9.08	194.25	14.03	0.13
GD5	8.39	1532.60	6.74	194.10	4.95	0.28
GD6	8.76	733.38	7.10	183.15	4.39	0.22
GD7	8.26	3891.83	9.32	112.15	4.28	0.00
GD8	8.91	1760.58	4.96	21.30	56.83	0.10
GD9	8.30	1125.65	7.64	153.60	7.47	0.45
GD13	8.53	2338.15	6.37	184.90	11.56	0.08
GD14	8.16	3097.15	5.96	175.45	2.75	0.00
GD15	8.77	2903.55	6.49	67.85	7.98	0.04
GD16	7.93	1359.40	9.67	0.00	1.93	0.15
GD17	8.35	2427.18	8.62	95.60	34.50	0.19
GD18	8.36	2254.73	7.24	156.85	58.66	0.16
GD19	8.26	1749.78	8.50	207.70	3.58	0.36
GD20	7.93	1728.93	9.16	48.25	2.29	0.13
GD21	8.25	859.30	7.59	37.10	8.09	0.52
GD22	8.59	933.58	3.69	189.85	1.37	0.15
GD23	8.09	1969.58	6.23	59.05	2.42	0.00
GD26	8.82	1123.65	7.41	52.05	7.54	0.07
GD27	8.26	1703.70	11.74	19.70	9.27	0.00
GD28	8.03	4767.38	7.59	588.10	37.25	0.19
GD29	8.55	1142.48	5.73	74.00	45.49	0.11
GD31	8.24	3752.65	8.83	191.20	3.15	0.06
GD32	8.20	4281.85	10.83	137.90	21.43	0.13
GD33	8.23	2460.83	5.78	25.15	8.27	0.00
GD34	8.43	4236.90	7.27	122.60	17.86	0.15
GD36	8.70	3471.23	5.60	67.10	18.05	0.09
GD37	8.77	1090.30	5.05	29.35	67.77	0.08
GD38	8.26	1406.73	6.76	4.05	2.92	0.22
GD39	8.00	2534.75	8.28	142.75	5.00	0.13

**Table 3:** Sensitive and tolerant species for each stress factor.

	Tolerant Species	Sensitive Species
**∑TEQ_BaP_**	*Desulfuromonas_Desulfuromonas_*sp.	*Nitrospira_bacterium*
	*bacterium_YC-ZSS-LKJ56*	
	*Halomonas_xianhensis*	
	*Rhodovibrio_*sp.	
**TPH**	*Rhodovulum_*sp.	
	*bacterium_YC-LK-LKJ13*	
	*Bacillus_hwajinpoensis*	
	*Halomonas_ventosae*	
	*bacterium_YC-ZSS-LKJ56*	
	*Halomonas_xianhensis*	
	*Candidatus_Tectomicrobia*	
**EC**	*Rhodovulum_*sp.	*Palleronia_*sp.
	*Thalassobacillus_Thalassobacillus_*sp.	*Actinophytocola_*sp.
	*Candidatus_Tectomicrobia*	*Pontibacter_populi*

**Table 4:** Fitting results of different models for each stress factor.

Factors	ΣTEQ		TPH		EC	
Model	RMSE	HC_5_	RMSE	HC_5_	RMSE	HC_5_
Normal	0.0741	0.0639	0.1081	111.30	0.0520	892.072
Logistics	0.0663	0.0223	0.0816	150.00	0.0525	864.422

**Table 5:** Assigned EC_50_ values after modification.

Species	EC_50_ (Before)	EC_50_ (After)
*Bacterium_YC-LK-LKJ36*	0.06367	0.2005
*Anaerobic_bacterium*	0.06390	0.2329
*Bacterium_YC-LK-LKJ25*	0.07284	0.3298
*Bacillus_alkalitelluris*	0.1163	0.3532
*Bacillus_selenatarsenatis*	0.1194	0.4292
*Halomonas_ventosae*	0.1361	0.4586
*Blastococcus_*sp.	0.1588	0.7750
*Halophibacterium_profundimaris*	0.2108	1.580
*Bacterium_YC-ZSS-LKJ56*	0.2907	1.651
*Pseudomonas_borbori*	0.3904	2.798
*Gemmatimonadetes_bacterium*	0.5322	3.126
*Rhodovulum_*sp.	0.8769	3.785
*Tistlia_consotensis*	1.366	7.191
*Halomonas_xianhensis*	3.678	9.918
*Lysobacter_maris*	4.853	10.52
*Palleronia_*sp.	11.02	25.41
*Photobacterium_halotolerans*	17.17	39.47

**Table 6:** Regression equations of DF.

Regression Equations		R^2^
**n = 32**	DF = −1.073 SOM + 10.511	0.218
	DF = −1.132 SOM + 0.033 PAHs + 9.968	0.438

**Table 7:** Toxicity data of EC_50_ before and after the aging effects.

Species	EC_50_ (Before)	EC_50_ (After)
*bacterium_YC-LK-LKJ36*	0.2005	0.1956
*anaerobic_bacterium*	0.2329	0.2287
*bacterium_YC-LK-LKJ25*	0.3298	0.2801
*Bacillus_alkalitelluris*	0.3532	0.6575
*Bacillus_selenatarsenatis*	0.4292	1.074
*Halomonas_ventosae*	0.4586	1.188
*Blastococcus_*sp.	0.7750	2.246
*Halophibacterium_profundimaris*	1.580	2.892
*bacterium_YC-ZSS-LKJ56*	1.651	3.883
*Pseudomonas_borbori*	2.798	3.946
*Gemmatimonadetes_bacterium*	3.126	4.262
*Rhodovulum_*sp.	3.785	7.898
*Tistlia_consotensis*	7.191	10.16
*Halomonas_xianhensis*	9.918	12.95
*Lysobacter_maris*	10.52	24.31
*Palleronia_*sp.	25.41	45.36
*Photobacterium_halotolerans*	39.47	54.60

**Table 8:** Regression models of AF and the related environmental factors.

Regression Models		R^2^
**n = 32**	AF = 11.743 SOM + 42.867pH + 0.017EC	0.991
	AF = 242.518SOM +1256.029lgpH + 0.024EC − 1415.447	0.995

**Table 9:** Risk entropy and risk grade of the oil well site soil.

Sample	Aged Years	ΣTEF mg·kg^−1^	${RQ}_{\sum_{16}PAHs(NCs)}$	${RQ}_{\sum_{16}PAHs(MPCs)}$	Risk Level			
					RQ	P_i_	I_geo_	NIPI
GD1	21	0.18	626.82	0.13	1	5	3	5
GD2	33	0.02	58.01	0.01	1	5	3	
GD3	5	0.93	3319.46	0.71	4	5	4	
GD4	20	0.32	1142.69	0.25	4	5	3	
GD5	31	0.71	2532.71	0.54	4	5	3	
GD6	7	0.54	1944.23	0.42	4	5	4	
GD7	32	0.00	15.27	0.00	1	3	1	
GD8	20	0.26	933.64	0.20	4	5	3	
GD9	5	1.11	3974.25	0.85	4	5	3	
GD13	7	0.32	706.76	0.15	3	3	1	
GD14	7	1.30	9.82	0.00	1	5	3	
GD15	7	0.14	367.12	0.08	1	5	3	
GD16	7	0.33	1294.88	0.28	4	4	3	
GD17	11	0.16	1703.45	0.37	4	5	3	
GD18	11	0.32	1440.42	0.31	4	5	2	
GD19	15	0.89	3180.53	0.68	4	5	2	
GD20	15	0.36	1130.71	0.24	4	5	2	
GD21	15	0.01	4655.08	1.00	4	2	1	
GD22	20	0.48	1297.23	0.28	4	4	1	
GD23	21	0.20	22.54	0.00	1	5	3	
GD26	21	0.26	588.29	0.13	1	5	3	
GD27	21	0.21	33.12	0.01	1	2	1	
GD28	21	0.54	1719.15	0.37	4	5	2	
GD29	29	0.00	941.62	0.20	4	5	2	
GD31	30	0.10	502.79	0.11	1	5	3	
GD32	31	0.40	1129.17	0.24	4	5	4	
GD33	31	0.01	29.54	0.01	1	5	4	
GD34	31	0.37	1311.43	0.28	4	5	3	
GD36	31	0.22	799.30	0.17	3	5	3	
GD37	36	0.48	749.48	0.16	3	2	2	
GD38	39	0.01	1941.85	0.42	4	5	4	
GD39	41	0.36	1169.98	0.25	4	5	3	

## Data Availability

Data requests can be made to the first author.

## Supplementary Materials

The following supporting information can be downloaded at: https://www.mdpi.com/article/10.3390/toxics13050357/s1, Table S1: Chemical properties of the collected aged petroleum soils; Table S2: Toxicity equivalent coefficients and distribution coefficients of 16 PAHs; Table S3: Soil quality classification standards for a single pollution index; Table S4: Soil quality classification standard of the Nemerow index; Table S5. Classification standard of cumulative index method; Table S6: TEQ data after calibration.
