# Supplementary material for: Environmental DNA-Based Ecological Risk Assessment of PAHs in Aged Petroleum-Contaminated Soils
Source: Toxics. 2025 Apr 29;13(5):357. doi: 10.3390/toxics13050357 (PMC12115722; doi:10.3390/toxics13050357)
Supplement: Supplementary file 1 [file toxics-13-00357-s001.zip › supplementary materials .docx]

# Soil physicochemical properties

# The original data of physical and chemical properties of soil collected are shown in the following table.

**Table S1.** chemical properties of soil

| ID | Aged years | pH | EC/µs/cm | SOM/% | TPH（mg/kg） | ∑PAHs/ (mg/kg) | ∑TEF/（mg/kg） | ∑TEFw | 吸附校正后 |
| --- | --- | --- | --- | --- | --- | --- | --- | --- | --- |
| GD1 | 21 | 8.50675 | 2625.53 | 8.96 | 182.1500 | 4.07 | 0.1755 | 0.205057564 | 3.0764 |
| GD2 | 33 | 8.034 | 3261.60 | 7.56 | 48.7500 | 32.485 | 0.0162 | 0.035415219 | 0.4989 |
| GD3 | 5 | 8.3405 | 3932.00 | 7.64 | 37.7500 | 13.895 | 0.9294 | 1.692766587 | 0.5549 |
| GD4 | 20 | 8.189 | 4653.40 | 9.08 | 194.2500 | 26.735 | 0.3200 | 0.385740298 | 1.1878 |
| GD5 | 31 | 8.39 | 1532.60 | 6.735 | 194.1000 | 7.2875 | 0.7092 | 0.893292915 | 0.4711 |
| GD6 | 7 | 8.76 | 733.38 | 7.095 | 183.1500 | 6.3225 | 0.5444 | 1.120192074 | 1.6859 |
| GD7 | 32 | 8.262 | 3891.83 | 9.315 | 112.1500 | 8.55 | 0.0043 | 0.005475613 | 0.0091 |
| GD8 | 20 | 8.9065 | 1760.58 | 4.96 | 21.3000 | 113.25 | 0.2614 | 0.342749519 | 2.6222 |
| GD9 | 5 | 8.2975 | 1125.65 | 7.64 | 153.6000 | 11.055 | 1.1128 | 1.536311356 | 0.3885 |
| GD20 | 7 | 8.5305 | 2338.15 | 6.37 | 184.9000 | 3.46 | 0.3166 | 0.445617581 | 0.8589 |
| GD21 | 7 | 8.1555 | 3097.15 | 5.955 | 175.4500 | 10.69 | 1.3034 | 1.773010139 | 0.7836 |
| GD31 | 7 | 8.77 | 2903.55 | 6.485 | 67.8500 | 6.03 | 0.1408 | 0.247700712 | 0.5131 |
| GD39 | 7 | 7.9265 | 1359.40 | 9.67 | 0.0000 | 9.17 | 0.3276 | 0.456043564 | 0.0186 |
| GD26 | 11 | 8.35425 | 2427.18 | 8.615 | 95.6000 | 14.76 | 0.1647 | 0.348400183 | 1.6646 |
| GD32 | 11 | 8.36125 | 2254.73 | 7.235 | 156.8500 | 41.7675 | 0.3162 | 0.197662031 | 0.3806 |
| GD19 | 15 | 8.2645 | 1749.78 | 8.5 | 207.7000 | 4.0525 | 0.8905 | 2.519696699 | 0.0208 |
| GD22 | 15 | 7.926 | 1728.93 | 9.16 | 48.2500 | 1.365 | 0.3632 | 0.583545902 | 0.7057 |
| GD23 | 15 | 8.24975 | 859.30 | 7.585 | 37.1000 | 4.835 | 0.0063 | 0.012246393 | 1.6025 |
| GD28 | 20 | 8.58525 | 933.58 | 3.685 | 189.8500 | 73.615 | 0.4814 | 0.017496404 | 0.6042 |
| GD13 | 21 | 8.091 | 1969.58 | 6.23 | 59.0500 | 22.825 | 0.1979 | 0.356959236 | 0.2236 |
| GD29 | 21 | 8.8185 | 1123.65 | 7.41 | 52.0500 | 90.265 | 0.2637 | 0.924151634 | 2.6491 |
| GD37 | 21 | 8.3 | 1703.70 | 11.735 | 19.7000 | 134.995 | 0.2099 | 0.261248169 | 0.0517 |
| GD38 | 21 | 8.03325 | 4767.38 | 7.585 | 588.1000 | 3.91 | 0.5437 | 1.142226413 | 0.9468 |
| GD14 | 29 | 8.54625 | 1142.48 | 5.725 | 74.0000 | 5.5 | 0.0028 | 0.006362866 | 1.1623 |
| GD15 | 30 | 8.237 | 3752.65 | 8.83 | 191.2000 | 15.775 | 0.1028 | 0.120805538 | 0.2686 |
| GD18 | 31 | 8.2015 | 4281.85 | 10.83 | 137.9000 | 116.105 | 0.4033 | 0.758979926 | 1.0675 |
| GD27 | 31 | 8.227 | 2460.83 | 5.775 | 25.1500 | 18.545 | 0.0093 | 0.259326524 | 0.0098 |
| GD34 | 31 | 8.43375 | 4236.90 | 7.27 | 122.6000 | 34.505 | 0.3672 | 0.576838574 | 0.5138 |
| GD36 | 31 | 8.70325 | 3471.23 | 5.595 | 67.1000 | 35.55 | 0.2238 | 0.338042778 | 3.4102 |
| GD17 | 36 | 8.7715 | 1090.30 | 5.045 | 29.3500 | 67.6125 | 0.4770 | 0.590555056 | 0.5618 |
| GD33 | 39 | 8.2595 | 1406.73 | 6.76 | 4.0500 | 16.54 | 0.0083 | 0.012563462 | 0.5131 |
| GD16 | 41 | 8.00275 | 2534.75 | 8.275 | 142.7500 | 2.315 | 0.3626 | 0.496307357 | 0.9440 |
| Average |  | 8.356239063 | 2409.696094 | 7.4784375 | 118.8671875 | 29.93242188 | 0.361124141 | 0.583212134 | 0.9366 |

# Data Correction

## Partitioning Effect

The influence of the partitioning effect on the soil toxicity data was corrected using empirical formulas. The octanol-water partition coefficients of 16 polycyclic aromatic hydrocarbons (PAHs) in soil and the toxic equivalent factors (TEF) of various PAHs were collected, as shown in Table 1.1 below. According to the Karickhoff empirical formula, it is assumed that the adsorption of PAHs by soil mainly occurs through the organic carbon part in the soil, and it has similarities with the partitioning behavior of PAHs in the octanol-water system. The formula is as follows:

$${TEQ}_{BaP}=\Sigma{TEF}_{i}\times C_{i}$$

$$K_{d}=\frac{C_{s}}{C_{w}}$$

$$K_{d}=f_{oc}\times K_{oc}$$

$$K_{oc}=0.63K_{ow}$$

$$K_{d}=0.63\times f_{oc}\times K_{ow}$$

$$logK_{d}=alogK_{ow}+b$$

Where:

$K_{d}$——Soil-water partition coefficient;

$K_{oc}$——Organic carbon-normalized partition coefficient;

$K_{ow}$——Octanol-water partition coefficient;

$f_{oc}$——The content fraction of soil organic carbon, %;

C_s_——The concentration of PAH in soil, mg/kg;

$C_{w}$—— The concentration of PAH in water, mg/L;

**Table S2.** Toxicity Equivalent Coefficients and Distribution Coefficients of 16 PAHs

| PAHs | Abbreviation | Toxic equivalent coefficient  (TEF) | log K_ow_ |
| --- | --- | --- | --- |
| naphthalene | Nap | 0.001 | 3.37 |
| acenaphthylene | Acy | 0.001 | 4.08 |
| Acenaphthylene | Ace | 0.001 | 3.92 |
| Fluorene | Flu | 0.001 | 4.18 |
| Phenanthrene | Phe | 0.001 | 4.57 |
| Anthracene | Ant | 0.01 | 4.45 |
| Fluoranthene | Fla | 0.001 | 5.22 |
| Pyrene | Py | 0.001 | 4.88 |
| Benzoanthracene | BaA | 0.1 | 5.78 |
| Chrysene | Chr | 0.01 | 5.60 |
| Benzo [b] fluoranthrene | BbF | 0.1 | 6.04 |
| Benzofluoranthrene | BkF | 0.01 | 5.96 |
| Benzo [a] pyrene | BaP | 1 | 6.04 |
| Indenpyrene [1,2,3-cd] | IcdP | 0.1 | 6.56 |
| Dibenzoanthracene | DahA | 1 | 6.2994 |
| Benzo [g,h,i] perylene | BghiP | 0.01 | 6.36 |

## Aging Experiment Design

A total of 500 g of collected soil was weighed and placed in a glass beaker, sealed with Parafilm, and wrapped in newspaper to prevent moisture intrusion caused by water vapor. The soil was sterilized three times at high temperature, and after each sterilization, the soil was thoroughly mixed to ensure complete sterilization. After sterilization, the soil samples were cooled to room temperature. A 50 mL mixed solution of 16 polycyclic aromatic hydrocarbons (PAHs) in acetonitrile, with a concentration of 10 mg/L, was prepared using acetone and added to the soil. The mixture was thoroughly stirred and placed in a fume hood to allow the acetone to evaporate, resulting in contaminated soil samples with an additional PAH concentration of 1 mg/kg and a moisture content of 30% of the field capacity.The soil was then transferred to glass jars, covered with lids, and placed in a constant-temperature incubator. The incubator was set to simulate outdoor conditions with alternating day and night cycles: 28°C for 12 hours with 80% light intensity, and 20°C for 12 hours with 0% light intensity. Soil samples were collected at time points of 0, 7, 15, 30, 60, and 90 days. At each sampling time point, three soil samples were taken for the determination of PAH content, total petroleum hydrocarbons (TPH), soil organic matter (SOM), pH, and salinity.

# Risk assessment methods

The single Factor Pollution Index (Pi) is a relatively dimensionless index used to evaluate the level of soil, crop pollution or soil environmental quality, which can reflect the degree of pollution of various elements. The calculation formula is as follows:

$P_{i}=\frac{c_{i}}{s_{i}}$ (1)

Type: Pi - the ith element in the soil environment pollution index; Ci - measuring content of the ith element in soil (mg/kg); Si - the ith element evaluation standard of pollutants (mg/kg). Single factor pollution index method can reflect the pollution degree of each evaluation factor to facilitate comparative analysis between pollution factor, quickly judge the soil environment of the main pollution factor. This method has clear objectives and simple operation, but does not consider the toxicity of soil heavy metals. The actual soil environment is often the result of multiple factors, and it is mainly used to evaluate the specific area of single factor pollution. The grading standards are shown in **Table S3**.

**Table S3.** Soil quality classification standards for single pollution index

| Risk Level | Pi | Pollution condition |
| --- | --- | --- |
| 1 | Pi≤1 | Non-pollution |
| 2 | 1<Pi≤2 | Minor pollution |
| 3 | 2<Pi≤3 | Mild contamination |
| 4 | 3<Pi≤5 | Medium pollution |
| 5 | 5<Pi | Severe Pollution |

Nemerow Composite index method (NIPI) is one of the most commonly used methods to calculate composite pollution index at home and abroad. The method calculates the multiple of exceedance for each factor, and then calculates the average of each sub-index, taking the largest sub-index and the average. The calculation formula is as follows:

$P=\sqrt{\frac{{{(\bar{P}}_{i ave})}^{2}+{{(\bar{P}}_{i max})}^{2}}{2}}$ (2)

Type: P - inside, the comprehensive pollution index; Pi I ave - elements of average pollution index; Pi Max - element pollution index is the highest ratio of the I.

The pollution status is shown in the P-values calculated for the 5 pollution levels in **Table S4**.

**Table S4.** Soil quality classification standard of Nemerow index

| Risk Level | P | Pollution condition |
| --- | --- | --- |
| 1 | P≤0.7 | Non-pollution |
| 2 | 0.7<P≤1 | Minor pollution |
| 3 | 1<P≤2 | Mild contamination |
| 4 | 2<P≤3 | Medium pollution |
| 5 | 3<P | Severe Pollution |

Soil accumulation index method is used to assess the pollution levels of various pollutants in soil samples based on soil background values of pollutants and human activities. The calculation formula is as follows:

$$I_{geo}=\log_{2} \left( \frac{C_{n}}{{K\times B}_{n}} \right)$$

Type: 𝐼 𝑔 𝑒 𝑜 - earth cumulative index; 𝐶 𝑛 - concentration of pollutant in soil; 𝐵 𝑛 - pollutants geochemical background values of n. K - geological rock, sedimentary characteristics that result in the change of the background value coefficient of 1.5 (generally). General Igeo is divided into seven levels, geological accumulation index and pollution degree of classification as shown in **Table S5.**

**Table S5.** Classification standard of cumulative index method

| Risk Level | I_geo_ | Pollution condition |
| --- | --- | --- |
| 0 | I_geo_<0 | Non - pollution |
| 1 | 0≤I_geo_<1 | Non - Medium pollution |
| 2 | 1≤I_geo_<2 | Medium pollution |
| 3 | 2≤I_geo_<3 | Medium - Heavy pollution |
| 4 | 3≤I_geo_<4 | Heavy pollution |
| 5 | 4≤I_geo_<5 | Heavy - Extremely heavy pollution |
| 6 | 5≤I_geo_ | Extremely heavy pollution |

# Statistics of Calibration

**Table S6.** TEQ data after calibration

| ID | TEQ After adsorption correction | TEQ after aging correction |
| --- | --- | --- |
| GD1 | 3.076432639 | 8.447181081 |
| GD2 | 0.498858904 | 1.589745327 |
| GD3 | 0.554851736 | 120.5341887 |
| GD4 | 1.187804134 | 51.57133032 |
| GD5 | 0.471103169 | 28.15190082 |
| GD6 | 1.685943913 | 60.20334995 |
| GD7 | 0.009112866 | 0.545574571 |
| GD8 | 2.622214087 | 15.51808413 |
| GD9 | 0.388483212 | 56.13162982 |
| GD20 | 0.858872357 | 4.940732216 |
| GD21 | 0.783638564 | 0.310135475 |
| GD31 | 0.513122683 | 11.10398297 |
| GD39 | 0.018556393 | 15.4547491 |
| GD26 | 1.664577074 | 43.35835564 |
| GD32 | 0.380567564 | 87.36969001 |
| GD19 | 0.020833462 | 2.117916813 |
| GD22 | 0.705692798 | 0.904027781 |
| GD23 | 1.602452915 | 49.60141727 |
| GD28 | 0.604169519 | 4.356780276 |
| GD13 | 0.223598038 | 0.514206014 |
| GD29 | 2.649101356 | 4.284888854 |
| GD37 | 0.051657719 | 0.306453016 |
| GD38 | 0.946770902 | 61.91287076 |
| GD14 | 1.162297426 | 9.741947463 |
| GD15 | 0.268599024 | 6.17316083 |
| GD18 | 1.067520056 | 1.810206592 |
| GD27 | 0.009750613 | 0.413197768 |
| GD34 | 0.513829531 | 47.69062798 |
| GD36 | 3.410244199 | 11.41472603 |
| GD17 | 0.561847778 | 8.822524596 |
| GD33 | 0.513122683 | 113.0841876 |
| GD16 | 0.944038574 | 0.818458368 |

# Biological data

Biological data was shown in following <Species.xls>
